# QoS-Aware Cost Minimization Strategy for AMI Applications in Smart Grid Using Cloud Computing

**DOI:** 10.3390/s22134969

**Published:** 2022-06-30

**Authors:** Asfandyar Khan, Arif Iqbal Umar, Syed Hamad Shirazi, Waqar Ishaq, Mohsin Shah, Muhammad Assam, Abdullah Mohamed

**Affiliations:** 1 Department of Information Technology, Hazara University Mansehra, Mansehra 21120, Pakistan; asfandyar@hu.edu.pk (A.K.); arifiqbalumar@yahoo.com (A.I.U.); 2 Department of Telecommunication, Hazara University Mansehra, Mansehra 21120, Pakistan; waqarishaqk@gmail.com (W.I.); syedmohsinshah@hu.edu.pk (M.S.); 3 Department of Software Engineering, University of Science and Technology, Bannu 28100, Pakistan; soft.researcher12@gmail.com; 4 Research Center, Future University in Egypt, New Cairo 11835, Egypt; mohamed.a@fue.edu.eg

**Keywords:** advanced metering infrastructure, cloud computing, Internet of Things, latency, quality of service, scheduling, Smart Grid, virtual machine

## Abstract

Cloud computing coupled with Internet of Things technology provides a wide range of cloud services such as memory, storage, computational processing, network bandwidth, and database application to the end users on demand over the Internet. More specifically, cloud computing provides efficient services such as “*pay as per usage*”. However, Utility providers in Smart Grid are facing challenges in the design and implementation of such architecture in order to minimize the cost of underlying hardware, software, and network services. In Smart Grid, smart meters generate a large volume of different traffics, due to which efficient utilization of available resources such as buffer, storage, limited processing, and bandwidth is required in a cost-effective manner in the underlying network infrastructure. In such context, this article introduces a QoS-aware Hybrid Queue Scheduling (HQS) model that can be seen over the IoT-based network integrated with cloud environment for different advanced metering infrastructure (AMI) application traffic, which have different QoS levels in the Smart Grid network. The proposed optimization model supports, classifies, and prioritizes the AMI application traffic. The main objective is to reduce the cost of buffer, processing power, and network bandwidth utilized by AMI applications in the cloud environment. For this, we developed a simulation model in the CloudSim simulator that uses a simple mathematical model in order to achieve the objective function. During the simulations, the effects of various numbers of cloudlets on the cost of virtual machine resources such as RAM, CPU processing, and available bandwidth have been investigated in cloud computing. The obtained simulation results exhibited that our proposed model successfully competes with the previous schemes in terms of minimizing the processing, memory, and bandwidth cost by a significant margin. Moreover, the simulation results confirmed that the proposed optimization model behaves as expected and is realistic for AMI application traffic in the Smart Grid network using cloud computing.

## 1. Introduction

Recently, Smart Grid [1] has received great attention from researchers in the field of Information Technology (IT). The research community are developing new applications, communication protocols, and simulation models in order to add control, intelligence, automation, and communication capabilities to the existing traditional power grid system. For example, the new IT infrastructure in the Smart Grid paradigm consists of computing resources such as computation servers, storage servers, network devices, and Smart Grid applications [2,3], which are provided as services that perform fault tolerance, self-healing, demand response, load balancing, power generation, and optimal supply of electricity in an efficient and economic fashion in the Smart Grid network. Similarly, the intelligent smart meters (SMs) [4] at the customer domain (residential, commercial, and industrial) generate enormous metering data simultaneously to obtain real-time power consumptions, flexible pricing tariffs, and a monthly bill enquiry from the Smart Grid Utility provider. However, when the number of AMI application traffic increases between SMs and host servers in the control center, congestion occurs in the underlying Smart Grid network and performance degrades due to lack of sufficient computing resources in the existing IT setup. Using traditional techniques of centralized computing servers, storage, and database application, tightly coupled with business applications, leads to poor system reliability, higher cost, and time consumption in making a quick decision on the received real-time AMI application traffic in the Smart Grid network. Therefore, to fully realize the complex Smart Grid network in a cost-effective manner, the primary challenges are to improve the availability, reliability, and efficiency of the computing resources and quality of service (QoS) of application services, which are most important to be considered in the Smart Grid network.

In order to cope with these challenges, coupling the Internet of Things (IoT)-based networks [5,6] with cloud computing [7,8] will play an important role in the development of an improved Smart Grid architecture. With the induction of these two modern networking technologies, the residential users (here, SMs) will keep no concerns about the IT resource allocation (hardware, software) and application services (here, database application) because cloud computing primarily focuses on providing these services in a flexible and pay-as per-use manner over the Internet. Organization such as Smart Grid should pay attention to understand the current usage of cloud resources and increasing traffic rates (rising workloads) in order to efficiently utilize the actual cloud resources and applications (business operations). Such strategies enhance the overall network performance in terms of the agreed QoS and reduced costs (here, paid for cloud services), which are the key parameters being monitored by both the consumers and Utility provider in order to save money and time. Although, in the cloud environment, horizontal (e.g., adding more virtual machines) and vertical (increase size of the CPU, storage, RAM, and bandwidth) scaling are employed by organizations to handle the growing demands, ensure uptime, and optimize the network performance. For simplicity, here, the cost of cloud resource [9,10] means the leasing of cloud resources or paying for the use of cloud services, e.g., database applications, which consumers pays to the cloud provider. In Smart Grid, QoS provision with agreed terms and conditions, i.e., service level agreements (SLAs), are set at the time of the smart meter connection and installation.

For example, many public cloud providers, such as amazon web service (AWS), have a free tier micro instance called elastic computing cloud (EC2), which provides a limited set of cloud resources (services) free for 750 h per month for 12 months and do not charge all inbound traffics. However, AWS charges consumers for bandwidth on an hourly basis for outbound data transfers such as network-intensive workloads (services), such as IoT, real-time applications, and other public traffic such as video, audio, and gaming, due to which consumers pay high monthly bills. Therefore, the optimization of bandwidth is essential to increase the throughput, eliminate bottlenecks, and reduce substantially the majority of cloud computing costs both from the perspective of consumers and organizations.

Therefore, in this article, our main focus was to efficiently handle the cloud resources in order to minimize the cost of its usages and improve the QoS parameters of all intended AMI application traffic in the Smart Grid network. At this end, motivated by the aforementioned discussion, this article introduces a QoS-aware hybrid queue scheduling (HQS) model for AMI application traffic in the IoT-based Smart Grid network using cloud computing technology. Our proposed model particularly extended our previous work in [11], by focusing comprehensively on system implementation, simulation modelling, and cost reduction in terms of efficiently utilizing cloud resources and ensuring QoS provisioning to all AMI applications in the Smart Grid. The acronyms used in this articles are described in Table 1. In this context, following are some of the significant research contributions of this article:

1. The relay nodes in the network topology and the control central server in the Smart Grid network were programmed to differentiate and classify the AMI application traffic into periodic, normal, and time critical classes based on their packet size, latency, and priority level.
2. Our proposed optimization model employed three algorithms in order to allocate the resources in an optimized manner and schedule the AMI application traffic with co-existence to the public traffic in the Smart Grid communication network.
3. To achieve QoS levels and reliable communication, the proposed QoS-aware HQS model employed a combination of priority queue (non-preemptive) and first-come, first-serve scheduling techniques. A priority metric was assigned to each AMI traffic to prioritize transmission. The priority metric was computed based on the packet size, latency, and priority level of the AMI application, that is, smaller priority metric traffic had the highest priority in transmission to meet the latency requirement.
4. We developed an objective function that was mathematically formulated in order to optimize the cloud resources in such a way to minimize the total cost incurred in the usage of cloud services.
5. Finally, we analyzed and validated the efficacy of our proposed QoS-aware HQS model through extensive CloudSim simulation. The simulation results obtained demonstrate that the objective function is accurately implemented and successfully minimized the total costs in terms of CPU processing, RAM, and BW in the cloud computing environment.

The remaining article is ordered as follows. Section 2 elaborates on the related study about AMI applications, which particularly focused on AMI traffic management and scheduling schemes employed in the Smart Grid network. Section 3 briefly describes the problem statement. Section 4 describes in detail the proposed QoS-aware hybrid queue scheduling model, which includes the AMI application traffic model, network model, problem formulation, and proposed optimization model for AMI applications with an operational example. In Section 5, we analyze and validate the CloudSim simulation results. Lastly, the article is concluded and future work is described in Section 6.

## 2. Related Work

In recent years, due to the rapid development in IT and wireless technologies, that is, specifically the IoT-based network supported by the cloud computing environment, has received great attention from the research community. These networking concepts are particularly useful in Smart Grid infrastructure to allow things (e.g., SMs) and people in a residential area to exchange huge amounts of metering data with the Utility control center in the Smart Grid network. However, due to the resource constraint in the Smart Grid network, efficient utilization of CPU processing, memory, and network bandwidth with low cost is essential as well as to fulfill AMI applications requirements such as QoS, which is a challenging task in these infrastructures. Therefore, we present an in-depth literature review of existing research topics, covering the most important such as the background of AMI applications, traffic management, and resource allocation strategies in a cost-effective manner in the Smart Grid network.

For ensuring QoS through traffic scheduling in the Smart Grid network, multiple research efforts have been performed in the past. For instance, in our previous work [11], we proposed an IoT-based hierarchical clustering architecture for AMI applications in the Smart Grid network. We derived an objective function that was implemented via different algorithms to maximize network coverage, minimize the infrastructure cost, and eliminate bottleneck problems in the network topology. In addition, the simulation results depicted that the scheduling policy optimizes the use of CPU processing in terms of the execution time of AMI application traffic, which significantly enhances the QoS such as to maintain reliability and low delay in the Smart Grid network. In [12], the proposed model was used to classify different Smart Grid application traffics with different QoS requirements into five traffic classes based on service differentiation unit. In class 1, traffics were scheduled with non-preemptive priority queue discipline, while other classes were scheduled with the round robin scheduling scheme. Analytical and simulation results showed that QoS requirements, such as delay (mean waiting times), for different Smart Grid traffics were satisfied. Similarly, a traffic scheduling algorithm was proposed in [13], which classified the Smart Grid application traffic into two different classes, namely event-driven and fixed scheduling. The scheduling model works in two stage that is, in the first stage, the bandwidth is efficiently allocated to the traffic in the event-drive class, while the remaining bandwidth is assigned to the second class to ensure QoS levels such as latency and bandwidth allocation to other traffics. In order to release congestion and minimize packet loss ratio in multi-hop wireless mesh networks, the authors in [14] proposed a new scheduling technique based on random-switching, which uses the load balancing concept to schedule burst data in the metering data collection tree. The simulation results showed that the proposed scheme makes metering data collection, traffic distribution, and traffic forwarding processes more reliable and efficient in the network. The authors in [15] proposed a packet scheduling algorithm to handle metering traffic in outage conditions. The proposed scheme considered the hop count from the mesh node to the gateway node and queue length in the packet scheduling process. Numerical and simulation results showed that the network’s delay was minimized and that the overall network reliability was enhanced under emergency situations, such as the exchange of outage notifications in the network. In [16], the authors addressed the uplink bottleneck in long term evolution (LTE) networks. To solve this problem, a scheduling policy was employed that takes into account the channel quality, traffic priority, and packet delay in order to send periodic electricity consumption data of SMs, along with users real-time traffic such as voice calls. The proposed scheduler served a great number of user’s traffic with coexistence to smart metering traffic in the LTE networks. An optimization scheme in [17] was proposed for critical data such as critical faults with high arrival rates in the monitored environment in order to maintain low latency and high reliability, as well as to provide QoS to critical data with low energy consumption using wireless sensor networks (WSNs) for Smart Grid applications. Similarly, using WSNs in the Smart Grid network, the authors in [18] proposed a delay-aware cross-layer (DRX) and fair and delay-aware cross-layer (FDRX) algorithms for Smart Grid monitoring applications to minimize the delay and maintain lower collision rates in the communication channel. In [19], a backscattering green technology was proposed for information exchange and energy signals for wireless sensor battery recharge in Smart Grid. An asynchronous advantage actor critic (A3C) model using priority was designed to handle uplink resource allocation to increase the throughput in the network.

The use of the cognitive radio network (CRN) was investigated as a key wireless technology in [20,21,22,23] for traffic scheduling and the optimization of resources to reduce interference in the communication channel and optimize bandwidth usage in the Smart Grid network. In [20], the authors proposed a scheduling algorithm in order to increase network throughput and preserve priority and stringent delay parameters of real-time Smart Grid applications, such as transmission line monitoring in rural areas, using cognitive radio (CR) technology. In [21], a QoS-aware packet scheduling mechanism based on prioritization and classification in CRNs for Smart Grid applications was proposed to enhance the transmission quality of secondary users, that is, high-priority as well as increase the system utilization. Similarly, the work in [22] presented an Adam optimizer scheduling technique for Smart Grid applications using CR technology to reduce latency, increase network throughput, and obtain optimal system cost in the Smart Grid. The proposed scheduling technique was expressed as an objective function that is equal to the sum of throughput and latency utility functions. In addition, priority classes and sub-classes of Smart Grid applications based on their latency and throughput parameters were categorized. The scheduler maintains priority queues for traffic classes. In addition, a traffic scheduling technique using the priority of various Smart Grid applications, such as meter readings, multimedia sensing data, and control commands, was proposed in [23] for a CR-based Smart Grid network. The work specifically focused on CR channel allocation and Smart Grid traffic scheduling to solve the utility optimization problem in the system.

Different studies researching IoT applications in Smart Grid are presented in [24,25]. In [24], the authors proposed an IoT-based model for meter billing and energy monitoring applications in Smart Grid. The case study analysis explained that the proposed system design contributes to prevent electricity shortages and reduce power wastages. IoT-based applications have been comprehensively analyzed in [25] for Smart Grid and smart environments such as smart homes, smart cities, smart metering, etc. Nowadays, cloud environment [26,27,28,29] offers promising deployment models and services such as software, infrastructure, and hardware on user’s requests over the computer networks. In [26], authors showed the effectiveness and weaknesses of different scheduling and allocation algorithms in the cloud environment using a CloudSim framework. In [27], multiple resource allocation techniques were proposed in order to increase the QoS and efficiently utilize the cloud resources. In [28], a simulation model using CloudSim was defined to save time and money for researchers and organizations by reducing the energy and expense load in the cloud framework. Similarly, in [29], the authors presented a cloud-based simulation model using a CloudSim simulator for hosting Smart Grid applications to fulfill their processing, storage, and network requirements in the cloud infrastructure. Various virtual machine (VM) allocation policies have been analyzed, taking different VM parameters in order to make decisions for hosting Smart Grid applications in the cloud environment.

In the light of aforementioned discussion, we concluded that most of the previous research works as tabulated in Table 2 and are multi-objective; that is, the existing works in the literature are considered either to improve the QoS requirement or optimize the resources using various scheduling approaches for application -specific to the Smart Grid network. The work in IoT-based cloud computing for resource optimization in a cost-effective manner for AMI applications in Smart Grid is limited and needs the attention of researchers. We developed an optimization model based on IoT networking and cloud computing, which specifically focuses on AMI application traffic modelling, classification, prioritization, and traffic scheduling, using a hybrid queue scheduling scheme to achieve both cost minimization and QoS provisioning objectives in the Smart Grid network. The existing works in the literature do not address both the objectives in the Smart Grid network.

## 3. Problem Statement

In residential areas (urban), a large number of SMs are deployed at the consumer premises in the Smart Grid network, since SM-based applications generate a huge amount of metering data that need to be transmitted to the software-defined control center in a timely and reliable manner over the public IP-based network in the Smart Grid. However, the relay devices (here, SMs and DCs) used in the communication network have limited built-in resources (CPU, RAM, and BW) to accommodate such a high volume of traffic with required QoS levels in the communication network. For instance, power control commands (PCC) will be immediately transmitted within the recommended latency boundary (1 s) during peak load hours in the Smart Grid communication network; that is, it requires network access within a short time period, hence calling for priority-based transmission. In the absence of an active queue management mechanism in these devices, data packets will be dropped or even delayed in such a situation; thus, a queue contention solution is required. In addition, the available BW requires careful handling of the network both for end-users and AMI applications intending to improve the QoS levels. This problem is mathematically formulated in Equation (1) as follows:

(1) $$\overset{N_{\mathrm{SM}}}{\underset{i=1}{\sum }}\lambda_{i}\left(t\right)>\mu_{j}\left(t\right)\;\forall \;i\in N_{\mathrm{SM}},\forall \;j\in N_{\mathrm{CH}}\;\mathrm{or}\;\forall \;j\in \mathrm{CCS}$$

where $i=1\ldots \ldots N_{\mathrm{SM}}$ are the total SMs, $\lambda_{i}$ is the packet arrival rate (Poisson distribution) from SMs, and $\mu_{j}$ is the service rate (here, RAM of finite size, processing power, and limited BW), that is, exponentially distributed in these devices at time “t”. 

From the perspective of the cloud-based control center, the exchange of metering data (e.g., power consumptions) plays a vital role in making complex decisions to balance electricity generation and distribution in the overall Smart Grid architecture. Therefore, suitable resource handling and scheduling schemes are required for AMI application traffic in these devices that aim to ensure that needful buffer space and BW is available, such that congestion is avoided and dropping rates of data packets are minimized in peak load hours. Since cloud provides these services (resources) on a pay as-per use basis, cost minimization needs to be considered from the business perspective of both the end-user and organization.

Hence, in the light of the above discussion, we attempted to resolve the optimization problem through a QoS-aware HQS model aiming to ensure the required QoS levels and the efficient utilization and allocation of limited resources in a cost-effective manner during the processing of complex computations at the cloud-based CCS in the Smart Grid network.

## 4. Proposed QoS-Aware Hybrid Queue Scheduling (HQS) Model

**Figure 1:**
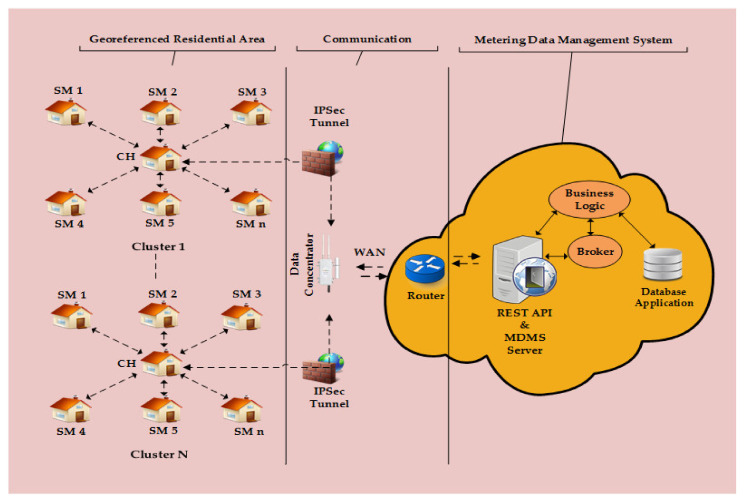
A simplified IoT and cloud-based hierarchical architecture of Smart Grid.

This section presents details about our proposed QoS-aware hybrid queue scheduling model for AMI applications in the Smart Grid network, which includes the sub-sections: AMI applications traffic model, network model with the main clustering topology (as shown in Figure 1), problem formulation, and proposed optimization model, such that resources are handled efficiently and the QoS requirements of each AMI application is maintained. A walk-through example is presented that clearly illustrates the working of the proposed optimization model in this article.

### 4.1. AMI Applications Traffic Model

The Smart Grid network consists of multiple applications such as Substation automation, demand response (DR), distribution automation (DA), and AMI, which consists of interval meter read (IMR), on-demand meter read (ODMR), electric vehicle (EV) charging, etc., where each has different traffic characteristics such as packet size, latency, data sampling frequency rate, reliability, and BW. Further, Smart Grid applications are classified into two different traffic types, namely deterministic and event-driven, as summarized in Table 3 below.

In this article, we only considered AMI applications from the SM perspective and presented how our proposed QoS-aware HQS model classifies various AMI-specific traffic (metering data) that is generated or received by the SMs into two different traffic classes, namely non-critical and time-critical, based on characteristics such as packet size, latency, and priority level. These characteristics may be pre-configured by the Utility administrator in all SMs and on other network devices before their deployment in the residential areas. The network model will support these traffic classes [3] with required communication characteristics, as listed in Table 4 below. Moreover, the non-critical traffic class consists of AMI applications such as IMR, ODMR, ODMRR, and billing information. IMR represents the customer electricity load profile (consumptions) typically sampled after every fixed time interval (e.g., 15–60 min), which can be mathematically expressed in Equation (2) as follows:

(2) $$\mathrm{IMR}=:\overset{n}{\underset{a=1}{\sum }}{\mathrm{kWh}}_{\mathrm{ah}}$$

where a=1,…, n. represents the household appliances, and ${\mathrm{kWh}}_{\mathrm{ah}}$ represents the electricity consumptions of an individual household appliance in kilo Watt per hour. The time interval depends upon the Utility provider. The IMR can be used later to prepare the consumer electricity bill, which may be shared with consumers through the billing information application. Similarly, ODMR is the meter reading request sent to the SM by the Utility provider, while on-demand meter reading response (ODMRR) is the SM response sent to the Utility provider that may be used in load forecasting and DR programs. On the contrary, the time-critical traffics consist of remote-control command (RCC), power control command (PCC), EV charging, and outage alert (OA) applications. Moreover, RCC includes commands such as remote disconnect\reconnect of devices, PCC consists of load control signals, EV charging shares electric vehicle charging information, and OA consists of messages such as outage detection sent to the Utility provider about the unavailability of electricity at the customer head-end. Among the two traffic classes, time-critical requires priority-based transmission due to its stringent latency and throughput requirements, while the non-critical could tolerate a delay of a few minutes in transmission in the Smart Grid network.

Once all the AMI applications are classified and their data sources (here, SMs) are identified, then the total traffic estimated for an AMI application can be expressed in Equation (3) [30] as:

(3) $$\mathrm{AMI}\;\mathrm{Traffic}\;\mathrm{Estimation}=:\overset{n}{\underset{i=1}{\sum }}\left({\mathrm{PS}}_{i}+{\mathrm{OS}}_{i}\right)\;\times \;{\left(N_{\mathrm{SM}}\right)}_{i}$$

where PS is the payload size, OS represents the overhead size of an SM, and $N_{\mathrm{SM}}$ is the number of SMs that generated the ith AMI application, whereas the traffic arrival rate $\lambda_{i}$ (Poisson process) at the ith device can be characterized [31] below as:

(4) $$\lambda_{i}=:\;\left\{\begin{array}{c} \rho_{i}\left(\lambda_{\mathrm{up}}+\lambda_{\mathrm{down}}\right)+\lambda_{\mathrm{up}}\;\mathrm{if}\;i\;\mathrm{is}\;a\;\mathrm{SM}\\ \rho_{i}\left(\lambda_{\mathrm{up}}+\lambda_{\mathrm{down}}\right),\;\mathrm{if}\;i\;\mathrm{is}\;a\;\mathrm{Router}\\ N_{\mathrm{SM}}\times \lambda_{\mathrm{down}},\;\mathrm{if}\;i\;\mathrm{is}\;a\;\mathrm{DC}\; \end{array}\right.$$

where $\rho_{i}$ represents the shortest path to the  ith device acting as relay node (here, CH), $\lambda_{\mathrm{up}}$ denotes the mean transmission rate from each SM to the DC (uplink), $\lambda_{\mathrm{down}}$ denotes the mean transmission traffic from the DC to each SM (downlink), and $N_{\mathrm{SM}}$ is the number of SMs connected to a DC in a given residential area. In addition, $\lambda_{\mathrm{up}}$ and $\lambda_{\mathrm{down}}$ can be calculated as:

(5) $$\lambda_{\mathrm{up}}\;\mathrm{or}\;\lambda_{\mathrm{down}}=\frac{{\mathrm{BW}}_{i}}{\;\mathrm{Pkt}\_{\mathrm{size}}_{i}}$$

where ${\mathrm{BW}}_{i}$ is the bandwidth (bits per second), and $\;\mathrm{Pkt}\_{\mathrm{size}}_{i}$ is the packet size of the ith AMI application. However, if the ith device retransmits the packet due to collision, then the actual traffic rate $\lambda_{i}^{⌁}$ [31] can be defined as:

(6) $$\lambda_{i}^{⌁}=:N_{i}{\times \lambda }_{i}$$

where $N_{i}$ is the average number of packets retransmitted at the ith device, and $\lambda_{i}$ is the traffic arrival rate as given in Equation (4). Another important factor that can be modelled is the mean service rate (here, BW) as given below:

(7) $$\mu_{i}=:\frac{{\mathrm{BW}}_{\mathrm{out}}}{{\mathrm{Pkt}\_\mathrm{size}}_{i}}$$

where ${\mathrm{BW}}_{\mathrm{out}}$ is the output BW allocated to an AMI application and is formulated as:

(8) $${\mathrm{BW}}_{\mathrm{out}}=:\alpha \overset{q}{\underset{i=1}{\sum }}{\mathrm{BW}}_{i}$$

where α is a constant factor between 0<α<1, and q represents the corresponding queue identifier that is allocated to each traffic class. Therefore, with an AMI traffic classification and traffic estimation (i.e., transmission rate) in hand, we needed to design an optimization model that will ensure the stringent QoS requirements (e.g., BW, latency, and throughput) and the reliability (e.g., accuracy and low packet errors) of each AMI application in the cloud-based Smart Grid network.

### 4.2. Network Model and Assumptions

The network model needed to be redesigned for the proposed model, which consists of smart meters (SMs) and data concentrators (DCs) at the lower level and a central router and main central control server (CCS) at the top level of the hierarchical IoT-based Smart Grid architecture. All these network components are pre-programmed (software defined) by the Utility operator in order to communicate with each other using default channel access methods in the underlined communication technology. A logical network topology for the proposed model is depicted in Figure 1 at the lower level in the hierarchical architecture. Further, the whole residential area was divided into IoT-based disjoint clusters using a modified K-means clustering method [11,32] (*though clustering is not the focus in this article*), where each cluster consists of a cluster-head (CH) and cluster-members (SMs). A CH operates similar to a traffic controller and scheduler to allocate resources and exchange the AMI application traffic between the cluster members and the DC in a single hop manner. In other words, each CH facilitates the communication between cluster-members and DC in a controlled manner to ensure the QoS levels of different AMI applications. The DC is directly connected over the Internet to the main CCS. For simplicity, we defined the total clusters as: K, cluster-members as: $N_{\mathrm{SM}}$, cluster-head as: $c_{K}$, number of active CHs as: $N_{\mathrm{CH}}$, and number of DC as: $N_{\mathrm{DC}}$ in the network model. Before discussing further, let us summarize the essential components of the network model as follows.

#### 4.2.1. Smart Meter

In Smart Grid, SMs [33] are typically installed at low altitudes in homes and buildings and play an important role in making decisions such as the demand and supply of electricity in Smart Grid. These SMs measure the power consumption of the home appliances at fixed-time intervals, as well as on demand, and exchange these measurements with the Utility provider via a shared communication network. Each SM has an IEEE 802.15.4g [34] interface used for intra-cluster communication as well as to enable the CHs to communicate directly with the DC. However, each SM has limited functionality due to built-in resources (CPU, RAM, and BW) in the Smart Grid network.

#### 4.2.2. IPSec Tunnel

The cluster members communicate locally and across the public IP network (Internet) with the main CCS using IPSec tunneling to ensure secure connectivity in the Smart Grid network. IPSec tunneling helps to provide security functions to AMI application traffic such as privacy and integrity, protection against non-repudiation, and replay attacks. Further, IPSec establishes secure connections via VPN between the pre-defined network devices (here, SMs, DCs, and the central router) and CCS of the Utility control center to limit the inbound and outbound traffic. VPN makes the IP address hidden which makes it harder for a flooding DDoS attack [35] to locate and target the Smart Grid network.

#### 4.2.3. Data Concentrator

Usually, DCs [36] are typically installed and mounted on top of poles in residential areas. The DC concentrates the metering traffic via CHs and forwards it to the main CCS for further storage and processing via the Internet. The DC has a higher processing capability, buffer space, and channel capacity and a longer radio range compared to the SMs in the Smart Grid network.

#### 4.2.4. Wide Area Network (WAN)

WAN interconnects the DCs to the main CCS via a bi-directional communication network that has a long-range communication and high network capacity (BW). WAN is the backbone network used to transmit the AMI application traffic, employing different technologies (e.g., optical fibre, wireless radio, LTE, etc.) [37], which have longer distance coverage and higher data rates. In this article, we opted to use LoRaWAN [38] as the WAN technology between the DCs and the central router at the control center head-end.

#### 4.2.5. Central Router

A central router, if present at the control center premise, facilitates the continuous exchange of metering traffic between the DCs and cloud applications deployed on top of the main CCS in the Smart Grid network.

#### 4.2.6. Control Center

The control center is a centralized component in the Smart Grid network that enables instant access to important resources (software and hardware) inside the Utility provider that creates a global view of the Smart Grid network (hierarchical architecture). This is why it is also termed the software-defined data center (SDDC) in the Smart Grid architecture. The control center includes a software system, referred to as the metering data management system (MDMS) [39,40], built on top of the main CCS, that performs complex computations (e.g., validation, analysis, and estimation) and stores the received metering data for long-term into a database application that contains data about the meters, their consumers, electricity bills, and other network devices. In particular, cloud services are deployed over the main CCS. To access the cloud services (e.g., database application), communication between the main CCS and SMs are established through specific RESTfull APIs over the Internet. Here, the message broker in the cloud framework manages the exchange of AMI application traffic (web request) between SMs and the database application. Further, necessary business logic and traffic rules (scheduling policies) are applied on received traffic that describe the clear scope and network behavior of the Smart Grid network. We made the following few assumptions in the design of our network model:

1. Only one DC at the centre of the residential area was considered.
2. Each device was assigned a unique IP address and meter registration ID.
3. Each device was authentic, and the CCS was fully trustworthy.
4. Every device was capable of computing the priority metric.
5. Finally, we considered that queuing delay was negligible, as we dealt with very low data rates.

### 4.3. Problem Formulation

Considering the optimization problem in Section 3, we focused on allocating a guaranteed number of resources such as CPU, RAM, and BW in a cost-effective manner; these are required by the AMI application traffic at the cloud-based CCS in order to ensure the QoS levels and improve the overall system performance of the Smart Grid network. The notations used in this section are already described in Table 1.

To optimize these limited resources, the optimization problem becomes a cost minimization problem. Therefore, the overall cost minimization problem can be formulated in the form of an objective function in Equation (9) as follows:

(9) $$\mathrm{minimize}\overset{K}{\underset{k=1}{\sum }}\overset{Q}{\underset{q=1}{\sum }}\overset{N_{\mathrm{SM}}}{\underset{i=1}{\sum }}{\left(C_{\mathrm{CPU}}+C_{\mathrm{RAM}}+C_{\mathrm{BW}}\right)}_{I,q}^{k}$$

Subject to:

(9a) $$\overset{K}{\underset{k=1}{\sum }}\overset{N_{\mathrm{SM}}}{\underset{i=1}{\sum }}\lambda_{k}^{i}\leq \mu_{k}^{i}\;\forall \;i\in N_{\mathrm{SM}},\;\forall {\;C}_{k}\in N_{\mathrm{CH}}$$

(9b) $$\overset{K}{\underset{k=1}{\sum }}\overset{Q}{\underset{q=1}{\sum }}\overset{N_{\mathrm{SM}}}{\underset{i=1}{\sum }}\frac{Q_{q}^{k}}{\lambda_{i}^{k}}\leq I\;\forall \;i\in N_{\mathrm{SM}},\;\forall {\;C}_{k}\in N_{\mathrm{CH}},\forall \;q\in Q$$

(9c) $$\overset{K}{\underset{k=1}{\sum }}\overset{3}{\underset{q=1}{\sum }}\overset{N_{\mathrm{SM}}}{\underset{i=1}{\sum }}{\mathrm{BI}}_{i,q}^{k}\leq {\mathrm{BW}}_{\mathrm{tot}}\;\forall \;i\in N_{\mathrm{SM}},\;\forall {\;C}_{k}\in N_{\mathrm{CH}},\forall \;q\in Q$$

(9d) $$\overset{K}{\underset{k=1}{\sum }}\overset{N_{\mathrm{SM}}}{\underset{i=1}{\sum }}{\mathrm{IW}}_{i,4}^{k}\leq {\mathrm{BW}}_{\mathrm{rem}}\;\forall \;i\in N_{\mathrm{SM}},\;\forall {\;C}_{k}\in N_{\mathrm{CH}},\forall \;4\;\in Q$$

(9e) $${\mathrm{BW}}_{\mathrm{rem}}\geq 0\;\forall \;i\in N_{\mathrm{SM}},\;\forall {\;C}_{k}\in N_{\mathrm{CH}},\forall \;4\;\in Q$$

In Equation (9), the total cost is expressed as a sum of each resource cost: CPU processing, RAM, and BW, where K represents the total number of clusters in the network model, $C_{k}$ denotes the corresponding CH, $N_{\mathrm{CH}}$ denotes the number of cluster heads, Q is the number of priority queues created at each device, and $N_{\mathrm{SM}}$ represents the number of cluster-members in each cluster. The objective function in Equation (9) intends to reduce the total system cost in terms of CPU processing ($C_{\mathrm{CPU}}$), buffer space ($C_{\mathrm{RAM}}$), and bandwidth ($C_{\mathrm{BW}}$) through the optimal allocation of these resources during the transmission of AMI applications in the Smart Grid network by ensuring constraints (9a)–(9e), whereas constraint (9a) ensures that the average traffic arrival rate ($\lambda_{i}^{k}$) at a device should be less than or equal to the average service rate $\mu_{i}^{k}$ at that device to minimize traffic losses or traffic retransmission later. Next, constraint (9b) satisfies that the AMI traffic is transmitted in the recommended latency ($L_{R}$) range in terms of the average traffic arrival rate and a particular queue length ($Q_{q}^{k}$), respectively. Constraint (9c) ensures that the guaranteed output BW is allocated to all AMI traffic in the corresponding queues (Q). Finally, constraint (9d) and (9e) ensures that the remaining portion of bandwidth (${\mathrm{BW}}_{\mathrm{rem}}$), i.e., non-negative, is allocated to other public traffic that exists in the communication network, where ${\mathrm{BW}}_{\mathrm{rem}}$ is expressed in (9f) below as:

(9f) $${\mathrm{BW}}_{\mathrm{rem}}={\mathrm{BW}}_{\mathrm{tot}}-\overset{K}{\underset{k=1}{\sum }}\overset{3}{\underset{q=1}{\sum }}\overset{N_{\mathrm{SM}}}{\underset{i=1}{\sum }}{\mathrm{BW}}_{i,q}^{k}$$

### 4.4. Proposed Optimization Model

In this section, we provide details about the proposed optimization model (QoS-aware HQS model) for AMI applications in this article that intends to guarantee the QoS levels and reliability of different AMI traffics so that costs incurred in the cloud resource usage are minimized and that the system performance is improved. As mentioned in Section 4.2, we particularly focused on implementing the resource allocation and scheduling techniques in CHs, DC, and the main CCS during the transmission of AMI applications. Assuming that these network devices have received the AMI traffic, there must be a mechanism to determine their arrival order, accommodate buffer space (RAM), and allocate BW on a priority basis before transmitting towards their destination in the Smart Grid network. Therefore, the following necessary operations would be performed on the AMI traffic:

- The received traffic is classified into two traffic classes based on their characteristics.
- Further traffic characterization is employed to handle BW allocation.
- Different queues (M\M\1) are created based on priority levels of these traffic classes, and AMI traffics are accommodated in these corresponding queues in a sorted order.
- A combination of queue scheduling schemes is used to serve AMI traffics based on their priority metric assigned in these queues.

where the priority metric ($P_{i,\;q}^{k}$) of the ith AMI application traffic in the qth queue at the kth device can be computed as:

(10) $$\mathrm{Priority}\;\mathrm{metric}\;\left(P_{i,\;q}^{k}\right)=:{\left(\mathrm{Pkt}\_\mathrm{Size}\;\times \;L_{R}{\;\times \;P}_{L}\right)}_{i}$$

where Pkt_Size represents the packet size, $L_{R}$ is the latency, and $P_{L}$ denotes the priority level of the ith AMI application. These traffic characteristics are listed in Table 4, and usually $P_{L}$ values are set by the network operator of the Utility provider. For clarity, $P_{L}$ assists in the creation of priority queues (qth) in order to avoid conflict of the queue allocation; that is, the queue contention of AMI application traffic requires priority-based transmission without delay and packet loss, whereas the priority metric ($P_{i,\;q}^{k}$) computed in Equation (10) above assists in scheduling the AMI traffic in priority queues. In the proposed optimization model, four queues (Q=1,…,4) were created in buffer space at each device. The incoming traffic from time-critical class applications such as RCC, PCC, EV charging, and OA were accommodated into queue1 (Q1), which has the highest priority level, while, traffic from the AMI applications such as ODMR, ODMRR, and billing information from a non-critical class were placed into queue2 (Q2), which has the 2nd highest priority level. Further, queue3 (Q3) was reserved for IMR application, which may tolerate delay up to a few minutes during transmission and has the lowest priority level 3 in the AMI traffics. Queue4 (Q4) was assigned to other public traffics. To serve these queues, we acquired a hybrid queue scheduling model which operated as follows: First, non-preemptive priority queue scheduling (NP-PQS) was applied to Q1 and Q2 using the priority metric for each AMI traffic to ensure that time-critical traffic will be served first ahead of non-critical traffic due to their tight latency boundary. Most importantly, NP-PQS is more useful for making decisions on the order of traffic arrivals, which determines the priority level, RAM, and BW allocation at the output link when congestion is experienced. In contrast, the FCFS scheduling method (default) was applied to Q3, as the priority metric of all AMI traffics are the same. Finally, other public traffic in Q4 were served after all AMI traffics were scheduled and served.

As discussed above, we presented three algorithms to bring in practice the proposed optimization model in order to solve the constraints of objective function. Below is the pseudo code used in Algorithm 1 to classify the incoming AMI application traffic listed in Table 4 at various devices in the hierarchical Smart Grid network.
**Algorithm 1: for classification of AMI applications traffic****Input**:$K,N_{\mathrm{SM}},\mathrm{Pkt},$ S1Begin2$\mathrm{insert}\mathrm{TC},\;\mathrm{ToA},\;\mathrm{and}P_{L}\mathrm{flags}\;\mathrm{into}\;\mathrm{application}\;\mathrm{layer}\;\mathrm{header}\;\mathrm{of}\;{Pkt}_{i}$3$\mathrm{set}\;n,\;i=0;j=1;\;;Q\left[4\right]\left[S\right]=\varphi ;R_{1\ldots 4}=F_{1\ldots 4}=-1;\mathrm{ctr}={\;}^{\prime }{\mathrm{start}}^{\prime };$4$L:\;\mathrm{while}\;\mathrm{ctr}\;!{=}^{\prime }{\mathrm{stop}}^{\prime }\;\mathrm{do}$5$\mathrm{read}\;\sum_{k=1}^{K}\sum_{i=1}^{N_{\mathrm{SM}}}{\mathrm{Pkt}}_{i,j}^{k}\mathrm{using}\;\mathrm{Equation}\;(5)$6$\mathrm{if}\;{\mathrm{Pkt}}_{i}.\mathrm{TC}={=}^{\prime }{\mathrm{Time}-\mathrm{Critical}}^{\prime }\;\mathrm{and}\;{\mathrm{Pkt}}_{i}.\mathrm{ToA}={=}^{\prime }{\mathrm{RCC}}^{\prime }\;\mathrm{or}{\;}^{\prime }{\mathrm{PCC}}^{\prime }\;\mathrm{or}{\;}^{\prime }{\mathrm{EV}\;\mathrm{charging}}^{\prime }\;\mathrm{or}{\;}^{\prime }{\mathrm{OA}}^{\prime }\;\mathrm{then}$7$P_{L}\leftarrow 1$8$\mathrm{else}\;\mathrm{if}\;{\mathrm{Pkt}}_{i}.\mathrm{TC}={=}^{\prime }{\mathrm{Non}-\mathrm{Critical}}^{\prime }\;\mathrm{and}\;{\mathrm{Pkt}}_{i}.\mathrm{ToA}={=}^{\prime }{\mathrm{ODMR}}^{\prime }\;\mathrm{or}{\;}^{\prime }{\mathrm{ODMRR}}^{\prime }\;\mathrm{or}{\;}^{\prime }\mathrm{Bill}\;{\mathrm{Info}}^{\prime }\;then$9$P_{L}\leftarrow 2$10$else\;if\;{\mathrm{Pkt}}_{i}.\mathrm{TC}={=}^{\prime }{\mathrm{Non}-\mathrm{Critical}}^{\prime }\;\mathrm{and}\;{\mathrm{Pkt}}_{i}.\mathrm{ToA}={=}^{\prime }{\mathrm{IMR}}^{\prime }\;then$11$P_{L}\leftarrow 3$12else13$P_{L}\leftarrow 4$14end if15$switch\;\left(P_{L}\right)\;\mathrm{do}$16$\mathrm{case}\;1:\;\mathrm{queue}\left({\mathrm{Pkt}}_{i},\;R_{2},F_{1},0\right)\;\mathrm{break};$17$\mathrm{case}\;2:\;\mathrm{queue}\left({\mathrm{Pkt}}_{i},\;R_{2},F_{2},1\right)\;\mathrm{break};$18$\mathrm{case}\;3:\;\mathrm{queue}\left({\mathrm{Pkt}}_{i},\;R_{3},F_{3},2\right)\;\mathrm{break};$19$\mathrm{case}\;4:\;\mathrm{queue}\left({\mathrm{Pkt}}_{i},\;R_{4},F_{4},3\right)\;\mathrm{break};$20end switch21get ctr22end whie23$void\;\mathrm{queue}\;\left(\mathrm{Pkt},\;R,\;F,\;n\right)\;then$24if R >=S then25Display “Queue Overflow”26else27if R==−1 then R=028$Q\left[n\right]\left[R\right]=\;\mathrm{Pkt}$29R++30**if**F==−1thenF=031end if32$Return\;Q\left[4\right]\left[S\right]$33End$\mathrm{Output}:\mathrm{Save}\;\mathrm{incoming}\;\mathrm{packet}\;{\mathrm{Pkt}}_{i}\;\mathrm{in}\;\mathrm{corresponding}\;\mathrm{Queues}$

Algorithm 1 begins with the assumption that three flags are added to the application layer header in each packet using Line 3. In line 4, certain variables are initialized, including a two-dimensional array ($Q\left[4\right]\left[S\right]$) where four rows are created for four priority level queues, and S represents the queue length in the buffer space. The incoming packets (Pkt) are read in the next line of code according to the arrival pattern as defined in Equation (5) until the loop condition remains true. Lines 6–14 are used to classify and assign the priority level to each traffic class. This traffic differentiation can be conducted through the use of traffic class (TC) and type of application (ToA) flags, which were already added in the packet header. Next, incoming packets are placed into their respective queues based on priority level ($P_{L}$) and front (F) and rear (R) pointers in unsorted form using Lines 15–31. Finally, Algorithm 1 ends and returns the priority queues as output in the next lines of code. Once traffic classification and queue formation are completed, next we present Algorithm 2 to transmit and process both traffics (AMI and public) from these queues through queue scheduling schemes (NP-PQS and FCFS) using the priority metric. The pseudo code in Algorithm 2 is given below:
**Algorithm 2: for prioritization and transmission of AMI applications traffic****Input**:$Q\;\left[4\right]\left[S\right],\mathrm{Row},\;S,\;K,\;\mathrm{CH},\;\mathrm{DC}$1Begin2$set\;{\mathrm{Pkt}}_{i,j}=\varphi ;\;\mathrm{Row}=0;\mathrm{Col}=0;\mathrm{Prio}\_\mathrm{metric}=0;$3void SortPriorityQueue (Q[][],Row, Col) then4$for\;\left(i=0;i<Row;i++\right)\;do\;//\mathrm{only}\;\mathrm{first}\;\mathrm{and}\;\mathrm{second}\;\mathrm{row}\;\left(\mathrm{queue}\right)\;\mathrm{will}\;\mathrm{be}\;\mathrm{sorted}$5for (j=0;j<Col;j++) do6for (n=0;n<Col−j−1;n++) do7$Calculate\;\mathrm{Prio}\_{\mathrm{metric}\;}_{i,n}\;\mathrm{and}\;\mathrm{Prio}\_{\mathrm{metric}\;}_{i,n+1}\mathrm{using}\;Equ.10\;\mathrm{for}\;Q\left[i\right]\left[n\right]\;\mathrm{and}\;Q\left[i\right]\left[n+1\right]\;\mathrm{respectively}$8$If\;\mathrm{Prio}\_{\mathrm{metric}\;}_{i,n}>\mathrm{Prio}\_{\mathrm{metric}\;}_{i,n+1}then$9$swap\;\left(Q\left[i\right]\left[n\right],Q\left[i\right]\left[n+1\right]\right)$10$else\;if\;\mathrm{Prio}\_{\mathrm{metric}\;}_{i,n}==\mathrm{Prio}\_{\mathrm{metric}\;}_{i,n+1}\;then$11Display “Do nothing and proceed to next queue item”12end if13end for;end for; end for14Call SortPriorityQueue (Q[][], 2, S)15$ifQ\left[\mathrm{Row}\right]\left[\mathrm{Col}\right]\;!=\;\varphi \;then$16$if{\;\mathrm{Con}}_{\mathrm{ST}}\;\to \;\mathrm{DC}{=}^{\prime }{\mathrm{Disconnect}}^{\prime }\;\mathrm{OR}\;!receive\;\left({\mathrm{Con}}_{\mathrm{RP}}\right)\leftarrow \mathrm{DC}\;then$17$send\;\left({\mathrm{Con}}_{\mathrm{RQ}}\right)\to \mathrm{DC}$18${\mathrm{CH}}_{k}\leftarrow receive\left({\mathrm{Con}}_{\mathrm{RP}}\right)$19$if{\;\mathrm{Con}}_{\mathrm{RP}}=={\;}^{\prime }{\mathrm{Allowed}}^{\prime }then$20$for\;\left(i=0;i<4;i++\right)\;do\;$21for (j=0;j<S;j++) do22if i !=2 then23$\mathrm{NP}-\mathrm{PQS}(Q\left[i\right]\left[j\right])\to {\mathrm{Pkt}}_{i,j}$24$send\;\left({\mathrm{Pkt}}_{i,j}\right)\;\to \;\mathrm{DC}$25else26$\mathrm{FCFS}\left(Q\left[i\right]\left[j\right]\right)\to {\mathrm{Pkt}}_{i,j}$27$send\;\left({\mathrm{Pkt}}_{i,j}\right)\;\to \;\mathrm{DC}\;\mathrm{using}\;Equation\;\left(7\right)$28end if29${\mathrm{Allocate}\;\mathrm{bandwidth}\;\mathrm{BW}}_{\mathrm{out}}\;\mathrm{using}\;Equation\;\left(8\right)$30$Q\left[i\right]\left[j\right].\mathrm{remove}()$31end for;end for32${\mathrm{CH}}_{k}\leftarrow receive\;\left({\mathrm{ACK}}_{\mathrm{RP}}\right)$33$\mathrm{send}\left({\mathrm{Con}}_{\mathrm{TR}}\right)\to \;\mathrm{DC}$34else35$\mathrm{Retry}\;\mathrm{or}\;\mathrm{wait}\;\mathrm{for}\;{\mathrm{CH}}_{k}\mathrm{until}\mathrm{TTL}==0;\mathrm{using}Equation\left(6\right)$36end if37else38Display “Queue is empty”39Return Null;40end if41$Return\;{\mathrm{Pkt}}_{i,j}$42End**Output:** Save outgoing packet ${\mathrm{Pkt}}_{i,j}$ in the corresponding output interface 

Algorithm 2 begins with the initialization of necessary variables in Line 2. Before the transmission of AMI application traffic, Lines 3–14 are used to sort out the first two queues in ascending order using the bubble sort method using the priority metric. However, if more than one data packet has the same priority metric, then they are processed in the order in which they have arrived in these queues. Next, if the queue is not empty, using Line 15, a TCP\IP connection setup is initiated and established between the CH and DC in Lines 15–19 using notations such as connection status (${\mathrm{Con}}_{\mathrm{ST}}$), connection request (${\mathrm{Con}}_{\mathrm{RQ}}$), and connection reply (${\mathrm{Con}}_{\mathrm{RP}}$). First, the time-critical traffic from queue-1 is scheduled and processed, and then queue-2 is scheduled with NP-PQS, respectively. Similarly, queue-3 with IMR traffic and queue-4 with public traffic is scheduled with FCFS scheduling, respectively. The output bandwidth is allocated to the outgoing packet in Line 29. After successful transmission acknowledged with the (${\mathrm{ACK}}_{\mathrm{RP}}$) message, the packet is removed from the corresponding queues, and the connection is terminated with connection termination (${\mathrm{Con}}_{\mathrm{TR}}$) in Lines 30–33. However, if the connectivity fails, the CH request will be either stored in the buffer state until it is time for the live (TTL) session to expire, or it will try again using Lines 35–36. The algorithm returns nothing (null value) in lines 38–41 if the queue is empty. Algorithm 2 returns the outgoing packet as the output and ends in Line 41–42. Further, a similar procedure was adopted at the DC to transmit the AMI application traffic over the Internet towards the main CCS for further analysis and processing. Lastly, Algorithm 3 is presented, which helps to simulate the proposed optimization model using the CloudSim framework at the control center side. The pseudo code is given below:
**Algorithm 3: for simulation of QoS-aware HQS model at the cloud-based CC server****Input:**$K,N_{\mathrm{DC}},N_{\mathrm{SM}},\mathrm{Pkt},\mathrm{Row},S,\mathrm{RAM},\mathrm{HDD},\mathrm{BW}$1Begin2$set\;n=i=j=0;Q[\mathrm{Row}][S]=\varphi ;\mathrm{CloudletList}=\mathrm{HostList}=\varphi ;\mathrm{ctr}{=}^{\prime }{\mathrm{start}}^{\prime };\mathrm{pesNo}=1;$3$while\;\mathrm{ctr}\;!{=}^{\prime }{\mathrm{stop}}^{\prime }\;do$4$read\;\sum_{k=1}^{K}\overset{N_{\mathrm{DC}}}{\underset{j=1}{\sum }}\overset{N_{\mathrm{SM}}}{\underset{i=1}{\sum }}{\mathrm{Pkt}}_{i,j}^{k}\mathrm{using}\;Equation\;\left(5\right)$5call Algorithm 1 to classify and queue the incoming packets6get ctr7end whie8call Algorithm 2 and use Lines 2–14 to sort queue 1 & 2 respectively9$ifQ\left[\mathrm{Row}\right]\left[S\right]\;!=\;\varphi \;then$10$\mathrm{HostList}=\mathrm{new}\;\mathrm{Host}\left(\mathrm{HostID},\mathrm{RAM},\mathrm{HDD},\mathrm{BW},\mathrm{PE}\left(\mathrm{mips}\right),\mathrm{new}\;\mathrm{VMSchedularSpaceShared}\left(\mathrm{PE}\right)\right)$11$\mathrm{Datacenter}=\mathrm{new}\;\mathrm{Datacenter}\left(\mathrm{Name},\mathrm{Arch},\mathrm{OS},\mathrm{VMM},\;\mathrm{new}\;\mathrm{VMAllocationPolicySImple},\left(\mathrm{HostList}\right),\mathrm{HDD},0\right)$12$\mathrm{Broker}=\mathrm{new}\;\mathrm{DatacenterBroker}\left(“\mathrm{Broker}”,\mathrm{BrokerID}\right)$13VMList=new VM(VMID,mips,BrokerID,size,RAM,BW,pesNo,VMM, new CloudletSchedulerSpaceShared())14$\mathrm{Broker}\;\leftarrow \mathrm{VMList}\;\left(\mathrm{VM}\right)$15ifCloudletList== φthen16$set\;\mathrm{Name}\leftarrow \mathrm{Pkt}.\mathrm{ToA};\mathrm{Priority}\leftarrow \mathrm{Pkt}.P_{L}$17CloudLetList=New Cloudlet(ID,Name,pesNo,Length,Filesize,OutputSize,Priority,new UtilizationModelFull())18$repeat\;{\mathrm{Pkt}}_{n}\;\in Q\left[\mathrm{Row}\right]\left[S\right]\;do$19$Map:\;{\mathrm{Pkt}}_{n}\;\to {\mathrm{CloudLet}}_{n}$20${\mathrm{CloudLet}}_{n}\leftarrow \;\mathrm{BrokerID}$21${\mathrm{CloudLet}}_{n}\leftarrow \;\mathrm{VMID}$22$switch\;\left({\mathrm{CloudLet}}_{n}.\mathrm{Priority}\right)\mathrm{do}$23$\mathrm{case}\;1:\;\mathrm{NP}-\mathrm{PQS}\left({\mathrm{CloudLet}}_{n}\right)\;\mathrm{break};$24$\mathrm{case}\;2:\;\mathrm{NP}-\mathrm{PQS}\left({\mathrm{CloudLet}}_{n}\right)\;\mathrm{break};$25$\mathrm{case}\;3:\;\mathrm{FCFS}\left({\mathrm{CloudLet}}_{n}\right)\;\mathrm{break};$26$\mathrm{case}\;4:\;\mathrm{FCFS}\left({\mathrm{CloudLet}}_{n}\right)\;\mathrm{break};$27end switch28$\mathrm{CloudLetList}.\mathrm{add}\left({\mathrm{CloudLet}}_{n}\right)$29n++30Untill n≤ Q.Length−131else32$Display\;“\mathrm{Queue}\;\mathrm{is}\;\mathrm{empty}\;\left(\mathrm{nothing}\;\mathrm{to}\;\mathrm{process}\;\mathrm{on}\;\mathrm{server}\;\mathrm{CPU}\right)”$33Return Null;34end if35$\mathrm{Broker}.\mathrm{submit}\left(\mathrm{CloudletList}\right)$36start cloudsim.simulation()37stop cloudsim.simulation()38Return CloudletList39End**Output:** Save incoming packet in the corresponding Queues and show the output as CloudLetList (requests)

Algorithm 3 works in three parts to implement and simulate our objective function expressed in Equation (9). In the first part, important variables are initialized in Line 3, and incoming packets are read through Lines 4–8 until the loop condition remains true. Further, Algorithm 1 is called to classify and accommodate the AMI traffic into corresponding queues to satisfy constraint (9a). In the second part, Algorithm 2 is called in Line 9 to sort out the AMI traffic based on priority metric in the corresponding queues so that constraint (9b) is fulfilled. In the third part, Lines 10–38 are used to set up the cloud environment with scheduling algorithms (NP-PQS and FCFS) at the CCS to ensure constraints (9c)–(9e). The processed CloudletList is returned as output in Line 39, which shows that the optimization problem has been solved, i.e., resource utilization costs are minimized in an efficient manner, which results in saving money and time in the Smart Grid network.

### 4.5. A Walk-Through Example

**Figure 2:**
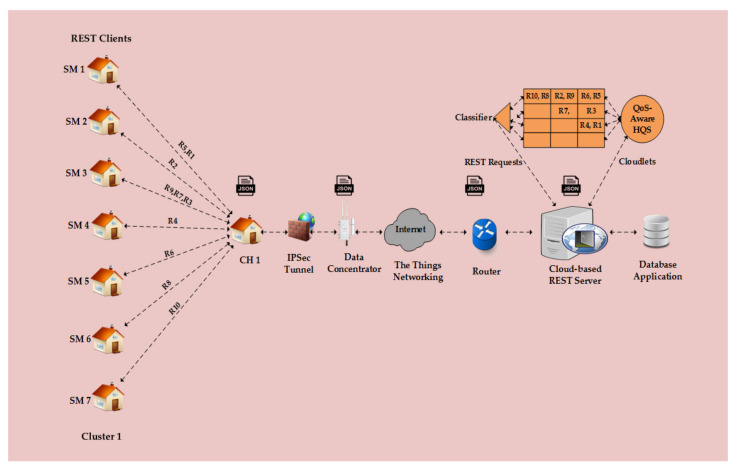
A walk-through example of QoS-aware HQS model using cloud computing.

In this section, we briefly present the working of our proposed optimization model to clarify the concept and validate the findings extracted from a small-scale residential area, as visualized in Figure 2. The IoT-based [33] Smart Grid hierarchical architecture is composed of SMs, DC, and the main CCS. In the clustering topology, SMs are configured to submit IMR data at fixed time intervals (e.g., 60 min) regularly and other metering data as needed to their respective CHs, which then transmit them to the DC. The DC forwards them to the main CCS over the Internet to access remote cloud services such as database application.

Therefore, an IoT-based architectural style (stateless client\server model) was adopted in designing the network model and applications via a set of RESTful APIs that are implemented using JavaScript-Node.js software. REST is a request-response IoT communication model, which is deployed to provide network connectivity to on-premise SMs and make available the cloud applications over the Internet as web services. This is why these SMs are also known as REST clients, which communicate with the main CCS, also referred as a REST server, using unique IP addresses in the TCP\IP network. The RESTful APIs support various HTTP methods such as POST, GET, PUT, and DELETE to manipulate a resource (software and hardware) of cloud environment. Multiple queries are passed (pushed) via a set of RESTful APIs (web services) [41] using HTTP protocol to exchange metering data in a standard format (JSON) between SMs and the main CCS with minimum efforts. Today, REST APIs are regarded as the “language of Internet”, as they are relatively easier to implement, test, and maintain, which makes them a better choice in the deployment of real world IoT communication models. Further, RESTfull APIs are language- and platform-independent, which works on top of HTTP-based standards and easily works with firewalls. Therefore, for any change in the firmware upgrade, configuration, etc., of the network devices, engineers need no manual intervention to change the parameters of the proposed model.

However, the devices in an IoT-based Smart Grid network are mostly resource-constrained, having limited CPU processing, RAM and BW capabilities, and different operational behaviour. REST architecture uses HTTP-based protocol, which requires extensive computational and memory capabilities to provide resources in the IoT-based Smart Grid network. Thus, it will be difficult for devices with limited resource capabilities to support HTTP-based RESTful APIs. Therefore, our proposed optimization model was employed aiming to optimize the resource allocation with QoS provisioning to AMI applications in the underlying IoT network. In the following example, we show that the metering data of seven SMs are listed in Table 5 and data were uploaded via a set of HTTP-based RESTful APIs methods to the cloud-based CCS (REST Server), as visualized in Figure 2. The received metering data were processed in real time and stored in a database application that was created in the Microsoft SQL server for future usages and analytics.

**Figure 3:**
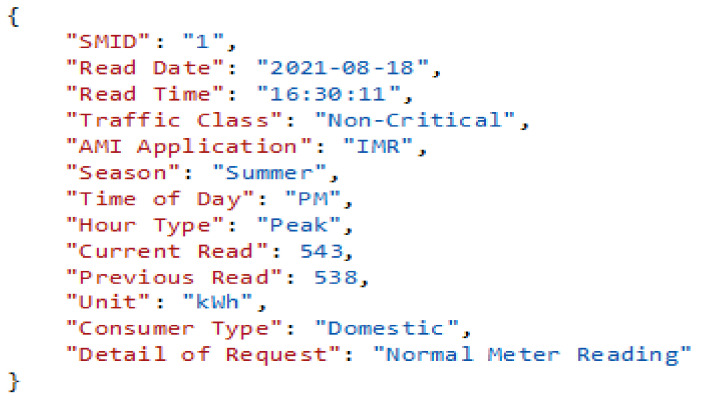
An example of the JSON packet generated by a smart meter (SM).

For instance, in Table 5 above SM1, (REST client) uses the HTTP-based POST method to submit request ($R_{1}$), that is IMR data in a JSON string (packet) with associated properties and values by creating the URL: (*http://192.168.1.1:9000/MeterReadAPI/NormalRead*) in a browser as shown in Figure 3 below.

Similarly, a RESTful API status line with code (HTTP\1.0 200 OK) in data acknowledgement (response) indicates that the POST request is received successfully at the cloud-based REST server. A different status code requires retransmission of the POST request. Now, the received requests are processed according to the proposed optimization model based on Algorithms 1, 2 and 3, respectively. The time-critical requests ($R_{2}$,$R_{5}$,$R_{6}$,$R_{8}$,$R_{9}$,$R_{10}$) having the highest priority level of 1 are stored in queue1 (Q1), and the non-critical requests ($R_{3}$, $R_{7}$) with the priority level of 2 are assigned to queue2 (Q2). Similarly, the remaining non-critical requests ($R_{1}$,$R_{4}$) are placed into queue3 (Q3), which has the lowest priority level of 3. Further, Q1 and Q2 are scheduled through the NP-PQS method based on the priority metric of each request, which is computed upon packet size, latency, and priority level, as expressed in Equation (10) and shown in Table 6 below. In addition, Q3 is scheduled on an FCFS basis. Public traffic is not considered in this example.

In this fashion, the resources are allocated in an optimized manner to these requests in order to ensure the QoS levels in terms of latency and throughput at the cloud-based REST server. The benefits of our proposed optimization model are that it is easier to design and can be deployed to any region in the Smart Grid network in a cost-effective manner.

## 5. Simulation and Performance Evaluation

This section presents details about the simulated cloud computing model for AMI applications by using the CloudSim simulator. The simulation results obtained show the correctness and effectiveness of our proposed optimization model.

### 5.1. Simulation Model

The CloudSim [26] simulator was used to simulate and estimate the performance of our QoS-aware HQS model as mathematically expressed in Equation (9). Further, CloudSim is a de facto and open-source Java-based platform (API) that allows the researcher and cloud developers in the simulation, experimentation, and modeling of the computing server (hardware) as Infrastructure as a Service (IaaS) [42] and services in the cloud environment. There are many cloud simulation tools, and among them, 18 [43] are extensions or derivatives of CloudSim. In addition, CloudSim incurs no installation and maintenance cost, is easy to use, is scalable, and helps to evaluate bottlenecks in earlier stages before deployment over the real-world cloud systems of both public and private cloud providers.

Since the cloud infrastructure provides “*pay as per usage*” services, it is necessary to optimize the resources in order to assess RAM, BW, and CPU processing for the control center to the SMs network communication. This can be accomplished via optimal resource allocation and scheduling algorithms in order to reduce money (cost) and time for organizations in cloud environment. The CloudSim classes are extended and modified for the proposed optimization model, and details are given in Table 7 below.

More precisely, a Data Centre was created and configured that has enough processing, storage, RAM, and BW capabilities to store and process metering data from multiple residential areas into a cloud-based database application. Further, Data Centre manages one host (physical server). Each host consists of a single or multiple CPU cores that are characterized by processer speed (i.e., MIPS), RAM, physical storage (i.e., HDD), and BW. An allocation policy in the Host decides how many CPU cores, CPU shares, and memory will be allocated to a designated VM. The Cloud simulation parameters are set on a personal laptop with configuration details, as given in Table 8 below.

One broker and two VMs were created for the Data Centre. The VM were assigned to the broker and each VM had the following configurations:

- VMM: “Xen”;
- VM RAM: 1 GB;
- VM processor speed: 300 MIPS;
- VM Scheduling policy: Time-shared;
- VM Image size: 512 MB;
- VM BW: 10 Mbps.

We created a pool of cloudlets that are defined in CloudSim as jobs\tasks in order to respond to specific incoming REST requests (AMI traffics) from REST clients. These incoming requests are managed by a broker with publish\subscribe protocols. Each cloudlet has the following configurations:

- File input size: 300 kb;
- Instruction length: Random (100–40,000);
- File output size: 300 kb.

We varied the number of cloudlets randomly according to the number of incoming REST requests that were received by the VMs. The CPU core allocation is managed by a VM Scheduler class that implements either the default time-shared or space-shared policy and can be modified to implement custom CPU allocation policies. The broker executes each cloudlet on VM according to a provisioning policy (e.g., space-shared) on resources of the physical Host in the Data Centre, since our cloud-based simulation model uses the CloudSim tool that requires a Java Runtime Environment (JRE) working in the cloud computing system. In addition, the CloudSim tool has no support for GUI, so an IDE such as Eclipse is required for the simulation model development in Java Language. Further, since CloudSim has no hardware constraint but a computer system with dual-core processing, 1 GB storage and 2 GB RAM will be good enough to run the simulation model with complex cloud scenarios. However, due to a lack of CloudSim support for distributed and parallel execution in memory systems, our simulation model is susceptible to support these execution techniques.

### 5.2. Simulation Results and Discussion

To evaluate the performance of our proposed optimization model (detailed in Section 4.4), we used a simulated cloud model. We used varying number of cloudlets in order to compute the cloud resources such as RAM, CPU, and BW demanded by each cloudlet. Moreover, the simulation results were quantified and compared to make sure that objective function developed for the AMI applications behaved as expected. After running the simulation model (detailed in Section 5.1), we obtained the following simulation results.

#### Objective Function: The Cost Minimization

During this simulation, we tried to validate the effectiveness of our proposed optimization model to achieve objective function, or the cost minimization, which is mathematically formulated using Equation (9) to demonstrate the optimal utilization in terms of RAM, BW, and CPU processing during the overall execution of cloudlets in the cloud environment. For this, in each simulation run, we took five sets with different ranges of cloudlets each consisting of 100, 200, 300, 400, and 500 cloudlets. In addition, each simulation run was carried over different settings of VMs and cloudlets having random lengths acquired by the queue scheduling schemes, i.e., FCFS [7], Priority-Based [8], and QoS-aware HQS in this article, whereas the cost incurred per cloud resource (CPU, RAM and BW) in each VM can be defined by the following three Equations (11)–(13), respectively.

(11) $$C_{\mathrm{CPU}}\left({\mathrm{VM}}_{i}\right)=\left(\frac{\mathrm{Actual}\;\mathrm{used}\;\mathrm{CPU}\left(\mathrm{MIPS}\right)\mathrm{by}\;\mathrm{Cloudlets}}{\mathrm{Total}\;\mathrm{CPU}\left(\mathrm{MIPS}\right)}{\mathrm{in}\;\mathrm{VM}}_{i}\right)*\mathrm{CostPerMIPS}$$

(12) $$C_{\mathrm{RAM}}\left({\mathrm{VM}}_{i}\right)=\left(\frac{\mathrm{Actual}\;\mathrm{used}\;\mathrm{RAM}\;\mathrm{by}\;\mathrm{Cloudlets}}{\mathrm{Total}\;\mathrm{RAM}\;\mathrm{capacity}}{\mathrm{in}\;\mathrm{VM}}_{i}\right)*\mathrm{CostPerRAM}$$

(13) $$C_{\mathrm{BW}}\left({\mathrm{VM}}_{i}\right)=\left(\frac{\mathrm{Actual}\;\mathrm{used}\;\mathrm{BW}\;\mathrm{by}\;\mathrm{Cloudlets}}{\mathrm{Total}\;\mathrm{BW}\;\mathrm{capacity}}{\mathrm{in}\;\mathrm{VM}}_{i}\right)*\mathrm{CostPerBW}$$

(14) $$C_{\mathrm{total}}\left({\mathrm{VM}}_{i}\right)=(C_{\mathrm{CPU}}+C_{\mathrm{RAM}}+C_{\mathrm{BW}})\;{\mathrm{in}\;\mathrm{VM}}_{i}$$

We set CostPerMIPS to 3.0 per second, CostPerRAM to 0.05 per megabyte, and CostPerBW to 0.1 per mega bit per second. Equation (14) presents the total cost, which is the sum of the CPU processing cost, RAM cost, and BW cost incurred during the scheduling and processing of cloudlets in VMs. The results obtained in each simulation run are tabulated in Table 9 below.

**Figure 4:**
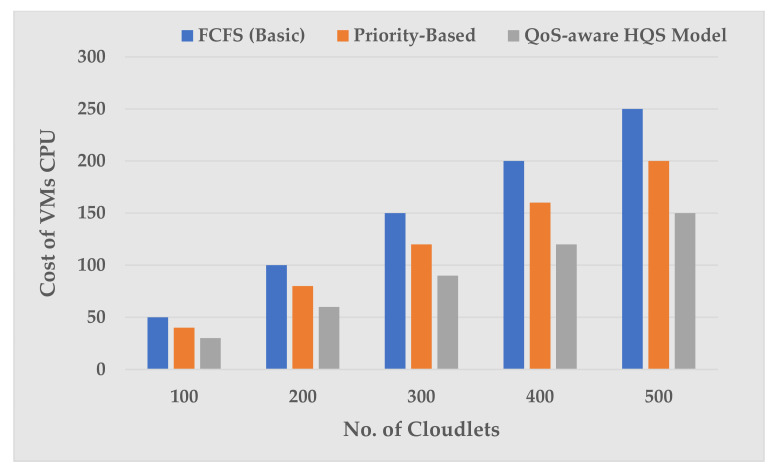
VMs CPU processing cost vs. number of cloudlets.

The simulation results shown in Figure 4 below are detailed above in Table 9. Figure 4 depicts the effect of different cloudlet ranges on the VM CPU processing cost. As the cloudlet size increases from 100 to 500, the cost of VM CPU processing is increased due to the number of instructions increased in cloudlets, i.e., more instructions will be transferred to VM CPU cores for processing. For example, for 100 cloudlets in 2 VMs hosted by 1 Host, the CPU processing cost incurred by FCFS is 50, while it is 40 in a Priority-Based scheme respectively. Similarly, the cost of CPU processing for 100 cloudlets by the QoS-aware HQS model is 30, which is less expensive than the other two scheduling algorithms. Further, we noticed the same less CPU processing cost with 200, 300, 400, and 500 cloudlets compared to other scheduling algorithms.

**Figure 5:**
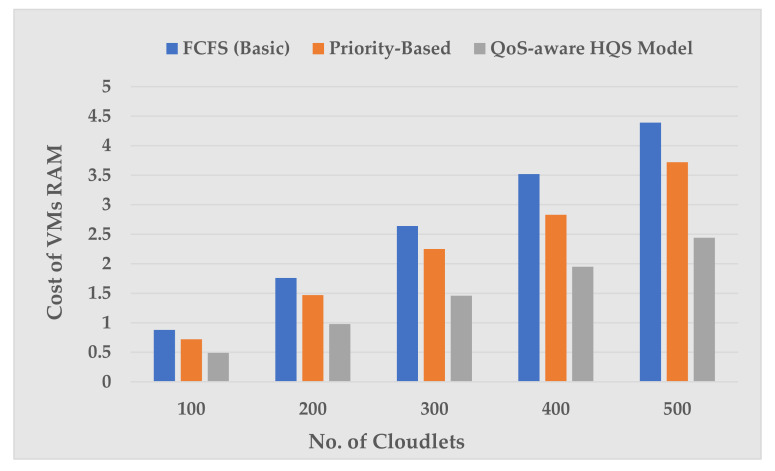
VM RAM cost vs. number of cloudlets.

Figure 5 renders the plot of VM RAM cost against a different set of cloudlets. The comparison of VM RAM cost incurred in the cloudlet execution reveals that the RAM cost was greater in the FCFS and Priority-Based scheduling scheme than our proposed scheme. For example, when the number of cloudlets are 100 (at RAM = 1024), the QoS-aware HQS scheme incurs 0.49 VMs RAM cost, which is 23.9% of the total VM RAM cost, as compared with the FCFS, which is 0.88 (42.92%), and Priority-Based is 0.68 (33.17%). Similar VM RAM cost trends have been deduced for cloudlet sets consisting of 200, 300, 400, and 500.

**Figure 6:**
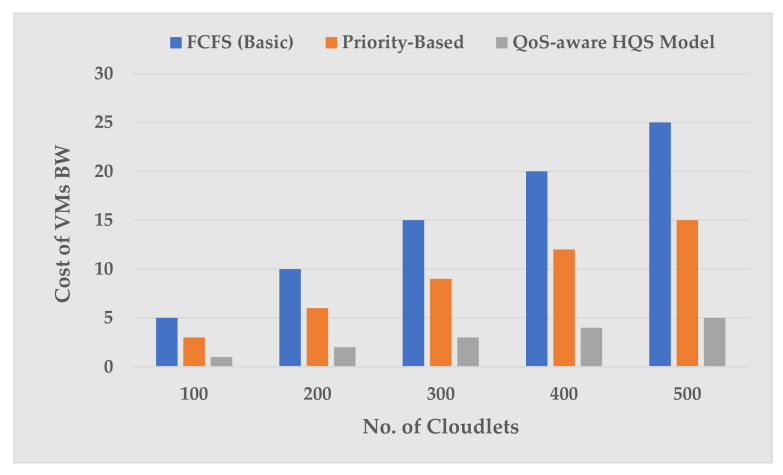
VMs bandwidth cost vs number of cloudlets.

Figure 6 plots the effects of VM BW cost on processing different sets of cloudlets. As the number of cloudlets increases, the cost of BW is much higher in the FCFS and Priority-Based scheme than in our proposed QoS-aware HQS scheme. The reason behind lower cost in terms of BW is that as we employed traffic classification and prioritization for AMI application traffic, which efficiently utilizes the BW and results into lower cost of BW. For example, at Cloudlets = 100, the BW cost of our proposed QoS-aware HQS scheme is 3× times lower than the Priority-Based and 5× times lower than the FCFS-based scheduling scheme. This is due to the FCFS and Priority-Based scheme sharing a single queue for all traffic, which leads to a queue contention problem. As a result, forwarding traffic flows may not be able to obtain enough BW, i.e., loss of BW occurs due to traffic contention, which increase the use of BW and its cost in resources. These simulation results confirm the effectiveness of our proposed scheme in this article.

**Figure 7:**
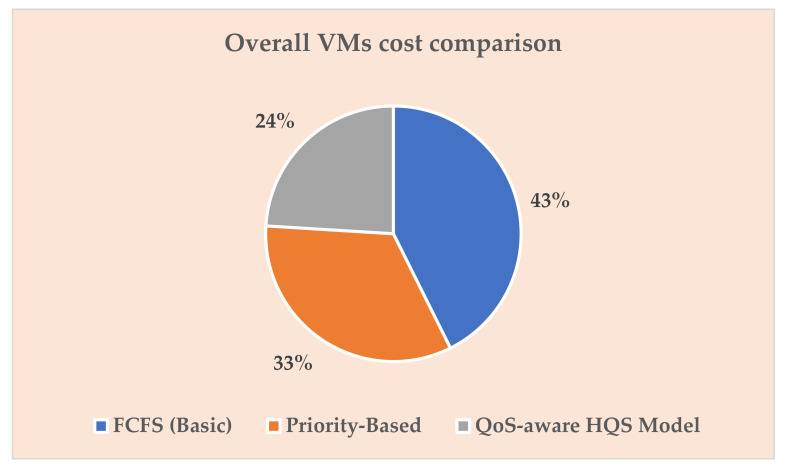
Overall VM cost comparisons.

Similarly, Figure 7 below depicts the overall cost comparison based on Equation (14) in percentile form, e.g., our proposed model significantly reduced cost to 24% compared to the other existing schemes, which shows its effectiveness and successful achievement of the objective function, as expected in the cloud-based Smart Grid network.

By comparing all these simulation results, we can deduce that our proposed optimization model efficiently utilized the cloud resources as expected, since it incurred lower costs in terms of CPU, RAM, and BW such that the cost minimization objective was successfully achieved and the target QoS requirements of all AMI application traffic were satisfied.

## 6. Conclusions and Future Work

In this article, we presented a novel optimization model for AMI application traffic in the Smart Grid network. The proposed optimization model relies on an IoT-based network coupled with cloud computing, which enables the Utility control center to remotely monitor and access the SMs in urban areas. SMs in the Smart Grid network generate a large amount of metering data (traffics) that heavily rely on adequate network infrastructure, including enough memory, storage, computing servers with higher processing, and bandwidth capabilities to cope with the target QoS requirements in the Smart Grid network. Therefore, we mainly focused on the optimal cloud resource allocations, which is the optimization problem. For this, we defined an objective function, a mathematical model, in order to reduce the costs incurred in terms of CPU processing, memory, and bandwidth during a wide number of system loads (cloudlets). To achieve this, we developed a QoS-aware HQS scheme which classified the AMI application traffic into two different traffic classes, namely time-critical and non-critical. In addition, the traffic from these classes were queued into four different priority queues, namely critical queue, normal queue, periodic queue, and public traffic queue with priority levels 1, 2, 3, and 4, respectively. Priority metric was computed based on the packet size, latency, and priority level of each traffic and was assigned to traffic in each queue. First, the NP- PQS scheme was used to schedule the traffic from the critical and normal queue, respectively, while the FCFS queueing discipline was applied to the periodic queue and public traffic queue, respectively. The proposed optimization model was implemented on a CloudSim simulator. Moreover, the efficiency and performance of our proposed optimization model was quantified and compared with other state-of-the-art scheduling scheme costs via simulation results. Finally, the simulation results confirmed that the proposed optimization model showed better cost reduction in terms of VM CPU processing, VM RAM, and VM BW on various lengths of cloudlets, as compared with the existing schemes. This cost evaluation is beneficial for researchers and organization in making decisions from a business perspective regarding deploying AMI applications on cloud frameworks, as it saves lots of time and money. At the end, we can deduce that the optimization model we developed for the AMI applications in the Smart Grid network behaves as expected.

Future Work—Our proposed optimization model integrates the emerging IoT technology with the cloud computing for client-server communication of AMI applications traffic in the Smart Grid network. The IoT framework is vulnerable to various security threats and becomes a challenging task to uncover malicious activities involving things such as consumers, SMs, DCs, and computing servers connected via the Internet in the world of the IoT-based Smart Grid. Therefore, lightweight security features are necessary to restrict malicious objects from establishing multiple connections with the network devices at a given time to avoid limited resource exhaustion (e.g., misconfiguration of firmware, etc.) and a DDoS attack. How our proposed scheme can address these security threats is a topic to be investigated in future work.

## Figures and Tables

**Table 1:** Acronyms and description.

Acronym	Description
AMI	Advanced metering infrastructure
API	Application programming interface
BW	Bandwidth
${\mathrm{BW}}_{\mathrm{tot}}$	Total available bandwidth
${\mathrm{BW}}_{\mathrm{rem}}$	Remaining bandwidth
CCS	Control center server
CPU	Central processing unit
$C_{\mathrm{BW}}$	Cost of bandwidth
$C_{\mathrm{CPU}}$	Cost of CPU processing
$C_{\mathrm{RAM}}$	Cost of random access memory
$C_{\mathrm{total}}$	Total cost
DC	Data concentrator
FCFS	First come, first serve
HDD	Hard disk drive
HTTP	Hypertext transfer protocol
HQS	Hybrid queue scheduling
IoT	Internet of Things
IPSec	Internet protocol security
JSON	Java script object notation
MIPS	Millions instructions per second
NP-PQS	Non-preemptive, priority queue scheduling
pesNo	Number of processing core
PE	Processing element
QoS	Quality of service
REST	Representational stateless transfer
SQL	Structured query language
SM	Smart Meter
URL	Uniform resource locator
VMM	Virtual machine manager
VPN	Virtual private network

**Table 2:** Summary of resource optimization strategies for AMI applications in the Smart Grid network.

Literature	Problem Specification	Resource Optimization Strategy	Applications (Traffic)	Network Environment	Objective (s)
[11]	Coverage Maximization Dual-head placement	Non-preemptive priority scheduling	AMI	IoT-based Cloud Computing	Improve QoS and coverage Minimize cost and Runtime
[12]	Modeling of Smart Grid Traffics	Non-preemptive priority and Round Robin	Smart Grid and VoIP	Smart Grid	QoS with low delay Increase throughput
[13]	Resource allocation approach	Two stage traffic scheduling	AMI	Smart Grid	QoS with latency requirement Optimize the BW
[14]	Network topology limitation	Random switching traffic scheduling	Smart Grid	Wireless Smart Grid	Minimize packet drop ratio Release congestion
[15]	Scheduling problem	Multi-gate and single-class back-pressure scheduling algorithm	AMI	Smart Grid	Improve network reliability and delay performance
[16]	Uplink bottleneck	Max rate uplink scheduler	AMI and real-time traffic	LTE-based Smart Grid	QoS with increased throughput
[17]	Delay optimization model	Inter cluster head scheduling	Smart Grid	WSN-based Smart Grid	QoS with low latency, energy consumption, and high reliability
[18]	Medium access approaches	DRX and FDRX data transmission	Smart Grid	Wireless actor and area network	Reduces delay Lower collision rate
[19]	Uplink resource allocation	Backscatter communication model	Smart Grid and energy	CR-based Smart Grid	Ensure business requirement Increase performance
[20]	Scheduling problem	Threshold triggered scheduling	Smart Grid	CRN	Maximize throughput Preserve priority and delay
[21]	Packet scheduling mechanism	Channel switch with priority scheduling	Smart Grid	CRN	Improves QoS and transmission quality
[22]	Multi objective optimization problem	Priority based queues using weights	Smart Grid	CR-based Smart Grid	Minimize latency Maximize throughput
[23]	Utility optimization problem	Priority based traffic scheduling and CR channel allocation	Smart Grid	CR-based Smart Grid	Low dropping rate Increases throughput
[24]	Energy meter billing and monitoring	Message queuing telemetry transport	Energy usages	IoT-based	Minimize energy wastage Prevent energy shortage
[25]	Energy internet based IoT application	A case study approach	AMI and Smart Grid	IoT systems	Highlight challenges and future opportunities of IoT applications in Smart Grid
[26]	CPU allocation and scheduling	Optimized Round Robin (RR) and FCFS	Cloud tasks	Cloud Computing	Shrink costs Increase IT quality
[27]	Optimum resource allocation	QoS based resource allocation (QRA)	Cloud request	Cloud Computing	Faster response time Low cost and energy
[28]	Resource allocation	Modified priority scheduling	Cloud applications	Cloud Computing	Save time and money
[29]	Simulation modeling of Smart Grid	Space-shared and time-shared VM policies	Smart Grid	Cloud Computing	Minimize cost of CPU, RAM, and BW
Proposed	Cost minimization strategy	Hybrid queue scheduling (HQS)	AMI	IoT-based Cloud Computing	QoS and cloud costs minimization

**Table 3:** Characteristics of Smart Grid applications [2].

Traffic Type	Smart Grid Application	Packet Size (Bytes)	Latency (Seconds)	Sampling Frequency (Per Day)	Bandwidth (Kb/s)	Reliability (%)
Deterministic	IMR	1600–2400	<4 h	4–6 (Residential) 12–24 (Commercial)	10–100 500 backhaul	99–99.99
Event-Driven	ODMR	100	<15	as needed	10–100	99–99.99
	EV charging	100	2 s–5 min	2–4	9.6–56	99–99.99
	Substation Automation	100	15–200 ms	as needed	9.6–56	99–99.99
	On-demand DR	100	500 ms–1 min	1 per event	14–100	99
	Real Time DR	100	500 ms–1 min	as needed	14–100	99
	DA	100	20–200 ms	as needed	9.6–100	99–99.99

**Table 4:** Characteristics of AMI applications [3].

Traffic Class (TC)	AMI Application (ToA)	Packet Size (Pkt_Size)	Latency $\left(L_{R}\right)$	Sampling Frequency (Per Day)	Traffic Type	Traffic Flow $(\lambda_{\mathrm{up}}$$/\lambda_{\mathrm{down}})$	Priority Level $\left(P_{L}\right)$
Non-critical	IMR	250 Bytes	5–60 min	12–24 (Residential) 4–6 (Commercial)	Deterministic	uplink	3
	ODMR	100 Bytes	<15 s	as per need	Event-driven	downlink	2
	ODMRR	100 Bytes	30 s	5 days		uplink	
	Billing info	100 Bytes	s or min	as per need			
Time-critical	RCC	100 Bytes	1 s	as per need	Event-driven	uplink	1
	PCC	100 Bytes	1 s	as per need			
	EV charging	100 Bytes	<15 s	2–4			
	OA	50 Bytes	3 s	as per need			

**Table 5:** An example of metering data exchanged via RESTful APIs.

SM ID	REST API	AMI Application	$\mathrm{Detail}\;\mathrm{of}\;\mathrm{Request}\;(R_{n})$	Date and Time	Season and Hour Type	Packet (Bytes)	Latency	Priority Level
SM 1	POST	IMR (Non-Critical)	$R_{1}$ = Current read 543	18 August 20211 6:30:11 PKT	Summer Peak	250	60 min	3
SM 2	POST	RCC (Time-Critical)	$R_{2}$ = Hardware Crash	18 August 2021 15:20:26 PKT	Summer Peak	100	1 s	1
SM 3	GET	ODMR (Non-Critical)	$R_{3}$ = Current Read?	18 August 2021 14:17:39 PKT	Summer Peak	100	<15 s	2
SM 4	POST	IMR (Non-Critical)	$R_{4}$ = Current read 820	18 August 2021 14:05:41 PKT	Summer Peak	250	60 min	3
SM 1	GET	RCC (Time-Critical)	$R_{5}=$ = Remote Disconnect	18 August 2021 13:27:54 PKT	Summer Peak	100	1 s	1
SM 5	POST	OA (Time-Critical)	$R_{6}$ = Outage Detection	18 August 2021 13:15:56 PKT	Summer Peak	50	3 s	1
SM 3	POST	ODMRR (Non-Critical)	$R_{7}$ = Current read 234	21 August 2021 12:31:16 PKT	Summer Off-Peak	100	30 s	2
SM 6	DELETE	RCC (Time-Critical)	$R_{8}$ = Hardware Crash	21 August 2021 12:31:18 PKT	Summer Off-Peak	100	1 s	1
SM 3	POST	OA (Time-Critical)	$R_{9}$ = Outage Restoration	21 August 2021 12:31:33 PKT	Summer Off-Peak	50	3 s	1
SM 7	GET	RCC (Time-Critical)	$R_{10}$ = OS Update	21 August 2021 11:44:13 PKT	Summer Off-Peak	100	1 s	1

**Table 6:** Output of the proposed optimization model.

SNO.	$\mathrm{AMI}\;\mathrm{Application}/\mathrm{Request}\;(R_{n})$	Priority Metric	Description
1	$R_{5}$	100	$R_{5}$ will be processed 1st
2	$R_{6}$	100	$R_{6}$ …………………… 2nd
3	$R_{9}$	100	$R_{9}$ …………………… 3rd
4	$R_{2}$	150	$R_{2}$ …………………… 4th
5	$R_{8}$	150	$R_{8}$ …………………… 5th
6	$R_{10}$	150	$R_{10}$ …………………… 6th
7	$R_{3}$	1000	$R_{3}$ …………………… 7th
8	$R_{7}$	6000	$R_{7}$ …………………… 8th
9	$R_{1}$	2,700,000	$R_{1}$ …………………… 9th
10	$R_{4}$	2,700,000	$R_{4}$ …………………… 10th

**Table 7:** Details of the simulated Cloud.

Cloud Classes	Value
Data Centre	1
Physical host	1
Broker	1
VM	2
Cloudlets	100–500

**Table 8:** Configuration details of the Host in a Data Centre.

Cloud Server	Configuration
Architecture	X64
Processor (CPU)	Intel(R) Core™ i5-8250M CPU@ 1.60 GHz 1.80 GHz
Processor speed (MIPS)	1000
RAM	8 GB
HDD	1000 GB
BW	50 Mbps
Operating system	Windows 10 Pro

**Table 9:** Cost of cloud resources in QoS-aware HQS Model compared with state-of-the-art scheduling schemes.

Simulation Run	Number of Cloudlets	FCFS (Basic) [7]			Priority-Based [8]			QoS-Aware HQS Model		
		CPU	RAM	BW	CPU	RAM	BW	CPU	RAM	BW
1	100	50	0.88	5	40	0.68	3	30	0.49	1
2	200	100	1.76	10	80	1.37	6	60	0.98	2
3	300	150	2.64	15	120	2.05	9	90	1.46	3
4	400	200	3.52	20	160	2.73	12	120	1.95	4
5	500	250	4.39	25	200	3.42	15	150	2.44	5

## Data Availability

Not applicable.
